# Shortage of Skilled Labor, Unions and the Wage Premium: A Regression Analysis with Establishment Panel Data for Germany

**DOI:** 10.1007/s12122-022-09334-1

**Published:** 2022-06-22

**Authors:** Arnd Kölling

**Affiliations:** Berlin School of Economics and Law, Alt-Friedrichsfelde 60, 10315 Berlin, Germany

**Keywords:** Shortage of skilled labor, collective bargaining, wage premium, classification, J23, J24, J51, J63

## Abstract

The author investigates the different influences of labor shortage on wages in firms with or without collective bargaining agreements. In addition to training, technological solutions, and organizational flexibility, employers can also offer higher wages at a constant employment level to fill vacancies if the current payments are lower than the marginal revenue of the workers. Firms with collective bargaining agreements probably already pay wages according to marginal revenue or, in the case of rent sharing, above it, and the remuneration is probably also not adjusted. Using wage regressions with panel data for German establishments, this paper shows that collective bargaining and a lack of skilled workers can lead to higher wages. However, the latter only applies to firms that are not bound by collective agreements. Hence, wage differentials between these firms decrease, providing further explanation for a countercyclical development of the wage premium from the collective bargaining agreement.

**Supplementary Information:**

The online version contains supplementary material available at 10.1007/s12122-022-09334-1.

## Introduction

With the outbreak of the COVID-19 pandemic, there are strong concerns in Germany about a potential shortage of skilled workers in the labor market. Prior to the pandemic, several companies reported significantly greater difficulties in filling their positions for skilled workers than in previous years. Since the great recession of 2009, the share of unfilled vacancies for skilled workers rose from approximately 16% to about 40% in 2018 (Dettmann et al., 2019). According to the KfW SME Panel, approximately 38% of companies with staffing problems cite “excessive wage demands” by applicants as the reason for not filling vacancies (Leifheit 2018). Similarly, difficulties in filling vacancies were frequently reported in areas that do not classify as requiring highly qualified experts. In Germany, skill shortages are often reported for occupations that correspond to skill levels 2 and 3 of the four-level scale of the International Standard Classification of Occupations (ISCO). These include, for example, a number of occupations in the construction industry and many healthcare professions that do not require academic training (Bossler et al. 2021; Federal Employment Agency 2020). It is also notable that pay structures have developed very differently in the sectors concerned. For example, according to the pay statistics of the Federal Employment Agency, the median wage of employees in the health sector rose by 16.1% in nominal terms between 2014 and 2018, while employees in the construction industries saw wages in their respective sectors rise by approximately 13.6% (Table 1). Moreover, the average changes of median wages in total is much lower, about 8.7%: This probably relates to the size of the reported skill shortage.

**Table 1:** Nominal Changes of Salary in selected industries (employees subject to social security contributions, 2014–2018, %)

	All industries	Health care and education	Construction
1. quintile	12.1%	20.3%	16.3%
2. quintile	9.5%	17.3%	13.6%
Median	8.7%	16.1%	13.6%
3. quintile	8.6%	15.0%	13.7%
4. quintile	8.9%	13.5%	14.1%
Share of employees covered by collective bargaining agreements	54%	60%	57%
Change of remuneration in collective agreements (2014 bis 2018)	10.4%	11.2%	10.9%

Source: Ellguth and Kohaut (2019), Federal Statistical Office Germany (Destatis), Federal Employment Agency Germany

Table 1 shows the wage increases for the different quintiles of the income distribution and those achieved through collective bargaining agreements. It can be seen that during the period considered, the lowest quintile, in particular, benefited from these increases. However, this is most likely due to the introduction of the statutory minimum wage in Germany in 2015. Moreover, since the table considers employees subject to social security contributions, this probably should not affect the median. Although the collectively agreed wage increases in the health and construction sectors are higher than in the economy in total, these were significantly smaller than the general wage increases for all quintiles in this sector; meaning that the wage premium of employees in firms with a collective bargaining agreement decrease. The opposite is the case when all industries are considered. Here, collective bargaining agreements cause an additional wage premium because wage changes of employees covered by collective bargaining agreements are larger than the average of all employees. This is in line with the observation of a countercyclical movement of the wage premium if labor shortage is related to additional economic growth in these industries. Usually, this is explained by unions protecting their members from decreasing wages in unfavorable market conditions (Freeman and Medoff 2018). In contrast, this analysis points to a mechanism based on wage increases in non-bargaining firms during an upswing.

This study, therefore, deals with the question of whether firms pay a wage premium when skilled labor shortages occur and the role of collective agreements in this context. Hence, the next sections contain an overview of theoretical considerations and empirical findings on the search for new employees from the employers’ perspective and the hypotheses derived. Data and the empirical model are presented, followed by the results of the regressions. A summary of the findings concludes the study.

## Review of existing literature

Various studies on the German job market show that employees paid according to collective agreements receive higher wages than comparable employees in companies not bound by such agreements (cf. Addison et al. 2014; Amlinger 2014; Gürtzgen 2016; Hirsch and Mueller 2020). Although these studies differ in the analyzed causes and the size of the wage premium, the figures in Table 1 indicate that in industries with skill shortages, the wage premium for a collective contract is lower. This confirms the empirical observation of a countercyclical wage premium when a shortage of skilled workers is linked to economic growth (Bratsberg and Ragan Jr. 2002; Blanchflower and Bryson 2004).

Regardless of whether a position is to be filled internally or externally, there are several ways for companies to overcome difficulties in hiring new personnel (Carrillo-Tudela et al. 2020). First, firms can probably increase their search and screening efforts to fill the open positions (e.g., Gavazza et al. 2018; Leduc and Liu 2020). Second, such positions might remain unfilled if the costs of searching become greater than the revenues generated when the position is filled, leading to developing alternative options such as labor-saving technologies (e.g., Andrews et al. 2008; Ehrenfried and Holzner 2019). Third, the position could be filled with applicants who do not (yet) possess the skills required. In the beginning, his productivity may not be as high as for a “suitable” applicant, and additional training costs may be incurred to provide the skills needed to fill the position optimally (e.g., Barron et al. 1997; Brenčič 2010; Sedláček 2014; Lochner et al. 2021). Finally, the company may try to make the job more attractive; for example, by offering a higher wage rate (e.g., Kaas and Kircher 2015; Schaal 2017; Banfi and Villena-Roldan 2019; Marinescu and Wolthoff 2020). Such wage offers are worthwhile for the company if it generates the lowest opportunity costs.

In addition to models that assume wage competition, there is also the possibility that the wage has already been determined and announced before the vacancy is filled (wage posting, cf. Manning 2011; Burdett and Mortensen 1998). Nevertheless, empirical studies have shown that wage negotiations are often under tight labor markets. By contrast, the rate of job offers with wage negotiations decreases if the applicant is unemployed and collective bargaining agreements are in place (Brenzel et al. 2014).

## Theory and Hypotheses

Considering a situation in which skilled workers are intensively sought for, perceived as a shortage of skilled workers, the probability of wage competition between firms should increase. Moreover, some employer search models initially assume that companies intend to offer the lowest possible wage at which an employee is willing to accept the job. However, this reservation wage must not be greater than the marginal revenue of the applicant (Cahuc et al. 2006). Wages will then fluctuate between the individual reservation wage and the marginal revenue of the workers in the firm. On average, wages paid are lower than the marginal revenue. If on-the-job searches take place and the employee receives a job offer from another company, the previous company can respond with a counteroffer. Following the model of Cahuc et al. (2006), I assume that the ability of workers is determined by ε. Here I consider labor shortages in specific occupations. Therefore, ε could be divided into two parts: the individual ability ε_i_ and the ability resulting from formal training in occupation ε_t_. While ε_t_ should be constant, ε_i_ is likely to be randomly distributed across workers with a mean of zero. The total individual ability ε_it_ is then given by:

1 $${\upvarepsilon}_{\mathrm{i}\mathrm{t}}={\upvarepsilon}_{\mathrm{i}}+{\upvarepsilon}_{\mathrm{t}}$$

Moreover, firms use different technologies, meaning the marginal revenue of efficient labor p is firm-specific. The marginal revenue of worker i in firm j is then given by ε_it_p_j_. A labor shortage is defined as a situation in which not all vacancies can be filled and unemployment in the particular labor market is negligible. Workers in firm j can immediately take advantage of job offers from other firms k with vacancies. When an individual receives a job offer, workers and employers negotiate wages. In the absence of outside job offers, an individual i is willing to work at their reservation wage ${\mathrm{w}}_{\mathrm{i}}^{\mathrm{r}}$. Formally, ${\mathrm{w}}_{\mathrm{i}}^{\mathrm{r}}$ are the discounted lifetime utility from receiving the reservation wage. Unlike Cahuc et al. (2006), I assume that workers and firms are likely to use hyperbolic discounting (see Ainslie and Haslam 1992) so that the current wage equals the lifetime utility. If ${\mathrm{w}}_{\mathrm{i}}^{\mathrm{r}}$ is lower than the marginal revenue ε_it_p_j_, the employer makes an additional profit by employing this worker: That is, ε_it_p_j_ is the maximum a firm is willing to pay. Thus, the negotiated wage lies between ${\mathrm{w}}_{\mathrm{i}}^{\mathrm{r}}$ and ε_it_p_j_, depending on the bargaining power of the workers and firms. The bargaining power of a worker is β (0 ≤ β ≤ 1) and the bargaining power of the firm is therefore (1 − β), the negotiated wage w_i_ is then:

2 $${\mathrm{w}}_{\mathrm{i}}={\mathrm{w}}_{\mathrm{i}}^{\mathrm{r}}+\upbeta \left({\upvarepsilon}_{\mathrm{i}\mathrm{t}}{\mathrm{p}}_{\mathrm{j}}-{\mathrm{w}}_{\mathrm{i}}^{\mathrm{r}}\right).$$

Without the support of unions, individual bargaining power is often assumed low and close to zero. Additionally, empirical estimations justify this assumption (Cahuc et al. 2006). Then, wage w_i_ depends on workers’ outside opportunities. If workers do not receive offers from other firms, then the wage is equal to the reservation wage:

3 $${\mathrm{w}}_{\mathrm{i}}={\mathrm{w}}_{\mathrm{i}}^{\mathrm{r}}.$$

The outcome is similar to a labor market that assumes monopsonistic structures and wage discrimination (Manning 2011). When workers receive job offers from other firms k, they must decide whether or not to leave the current employer. This should depend mainly on the level of wages offered ${\mathrm{w}}_{\mathrm{ik}}^{\mathrm{o}}$. Thus, the following situations may occur:

i. If ${\mathrm{w}}_{\mathrm{ik}}^{\mathrm{o}}$ < ${\mathrm{w}}_{\mathrm{i}}^{\mathrm{r}}$, the worker will stay at firm j without a raise.

If ${\mathrm{w}}_{\mathrm{i}}^{\mathrm{r}}$ < ${\mathrm{w}}_{\mathrm{ik}}^{\mathrm{o}}$ < ε_it_p_j_, the worker will stay at firm j but receives a raise of ε_it_p_j_ − ${\mathrm{w}}_{\mathrm{i}}^{\mathrm{r}}$. This term equals the difference between ${\mathrm{w}}_{\mathrm{ik}}^{\mathrm{o}}$ and ${\mathrm{w}}_{\mathrm{i}}^{\mathrm{r}}$.

If ε_it_p_j_ < ${\mathsf{w}}_{\mathsf{ik}}^{\mathsf{o}}$, the worker will leave firm j and accept the offer of firm k. The wage raise is still equal to the difference between ${\mathrm{w}}_{\mathrm{ik}}^{\mathrm{o}}$ and ${\mathsf{w}}_{\mathsf{i}}^{\mathsf{r}}$.

Assuming there is a labor shortage, i.e., the demand for certain workers is greater than the supply, the Bertrand-like competition drives up wages, and the firms with the lowest productivity are unable to fill vacancies because their maximum wages ε_it_p_j_ are lower than external offers ${\mathrm{w}}_{\mathrm{ik}}^{\mathrm{o}}$ and a further increase would hurt their profit-maximizing conditions. Then, applicants also demand wages deemed “too high,” and companies have to increase their productivity in order to pay wages following the market wage rates. Since marginal revenue depends on both the workers’ skills ε and firms’ productivity p, workers with the greatest skills in the most productive firms will receive the highest wages: this leads to a sorting process where employees with higher productivity work in more productive companies (Banfi and Villena-Roldan 2019; Brändle et al. 2020). Furthermore, companies with high productivity are then able to employ more workers, while companies with low productivity cannot fill their positions (see Burdett and Mortensen 1998). Moreover, workers are willing to change their firm if ε_it_p_j_ = (ε_t_ + ε_i_)p_j_ < ${\mathrm{w}}_{\mathrm{ik}}^{\mathrm{o}}$ < ε_i_p_k_.

Unions increase workers’ bargaining power and are, therefore, a source of a wage premium (e.g., Hirsch and Mueller 2020). Using a right-to-manage model (see Cahuc et al. 2014, 431ff.), collective bargaining leads to wages that equal marginal productivity. In our model, this means that wages equal ε_it_p_j_. This reduces the likely responses of workers in terms of job offers to:

i. If ${\mathrm{w}}_{\mathrm{ik}}^{\mathrm{o}}$ < ε_it_p_j_, the worker will stay at firm j without a raise.
ii. If ε_it_p_j_ < ${\mathrm{w}}_{\mathrm{ik}}^{\mathrm{o}}$, the worker will leave firm j and accept the offer of firm k.

This means there are no wage increases in companies with a collective agreement. Employees who seek higher wages must leave the company: This also applies to collective bargaining agreements that negotiate wages and employment when compensation is greater than marginal revenue (“efficient bargaining,” cf. Cahuc et al. 2014). Then ε_it_p_j_ must be replaced by the agreed higher wages. Moreover, if collective bargaining agreements force firms to increase their productivity p (e.g., Addison et al. 2013), these firms can recruit new workers or avoid employment turnover by raising employees’ wages. Empirical results have also shown that individual and collective bargaining have different efficiencies; at least, at the company level, collective bargaining is more efficient than individual wage negotiations due to lower transaction costs (Braakmann and Brandl 2020). In addition, individuals usually do not have significant bargaining power (Cahuc et al. 2006). In firms with a collective bargaining agreement that includes rent sharing with the workers, the wages are higher than the marginal revenue of the worker (Hirsch and Mueller 2020). In this situation, the firms have no incentive to fill a vacancy unless the agreement forces them to do so, as profits decrease hiring a new worker.

A company bound by a collective bargaining agreement will probably enter wage competition and pay wages higher than those agreed if the economic conditions change significantly after the negotiation. This is more likely to happen with regional or sectoral collective bargaining agreements than with agreements on a company level. These are better suited to local economic conditions (Jung and Schnabel 2011). Furthermore, it is often assumed that nominal wages are rigid at the bottom end (Branten et al. 2018; Peng et al. 2020) such that there is no corresponding decrease in wages due to an expired collective agreement or when there is no longer a shortage of skilled workers, although positive wage effects might be observed in these situations due to a shortage of skilled labor.

Considering all the above, the following research hypotheses were formulated:

i. Workers in firms with a collective bargaining agreement receive a wage premium.
ii. Shortage of (skilled) labor leads to an increase of wages in companies that are not bound by collective bargaining agreements.
iii. For a given level of employment, the wage premium of collective bargaining agreements decreases if a shortage of skilled labor occurs.
iv. Due to nominal wage rigidity, (positive) wage effects are more likely to be observed when collective bargaining agreements are introduced or when a shortage of skilled workers occurs for the first time in an establishment.

## Data and empirical model

The data used were taken from the IAB Establishment Panel and consisted of representative observations of German establishments from 2008 to 2018. The Institute for Employment Research of the Federal Employment Agency began collecting data from the IAB Establishment Panel in western Germany since 1993 and from the new federal states since 1996. However, the 2008 to 2018 period was chosen as some of the explanatory variables were only available from 2008, and 2018 was the last wave available at the time of this analysis. The population of the IAB Establishment Panel includes all German establishments with at least one employee subject to social insurance contributions. The survey involved a stratified random sample of 17 sectors, 10 employment size classes, and from 16 regions (federal states) of the population. The survey showed a very high response rate: over 70% to 80% for firms that participated more than once. However, the data were unbalanced as new establishments replaced the panel mortality with exits and non-response (Fischer et al. 2009). These data were supplemented by information from the Establishment History Panel, which provides the official data about the employment statistics at the firm level, providing detailed information on employee characteristics (Eberle and Schmucker 2017). It is possible to use observations from more than 100,000 establishments as more than 75,000 complete observations are available for the fixed effects regressions in total.

Since the IAB Establishment Panel was set up for the needs of the Federal Employment Agency, detailed information on the number and remuneration of workers, the composition of the workforce, its business policies and training activities constituted a major part of the survey. The endogenous variable used in the regressions was calculated as the log of wages per capita. The survey contained information about the monthly wage bill and the number of employees. The logarithm of the ratio of both variables was then used in the regressions, assigning part-time workers with a value of 0.5. Moreover, like all other nominal values in the regressions, wages were discounted by the producer price index. The covariate of major interest concerned hiring problems. The establishments were asked whether the firms had problems filling all vacancies. Two particular dummy variables were used to deal with this issue. The first dummy variable becomes 1 (and zero otherwise) if the firm has general problems with hiring enough workers, while the second becomes 1 if this is true for qualified workers.

**Fig. 1:**
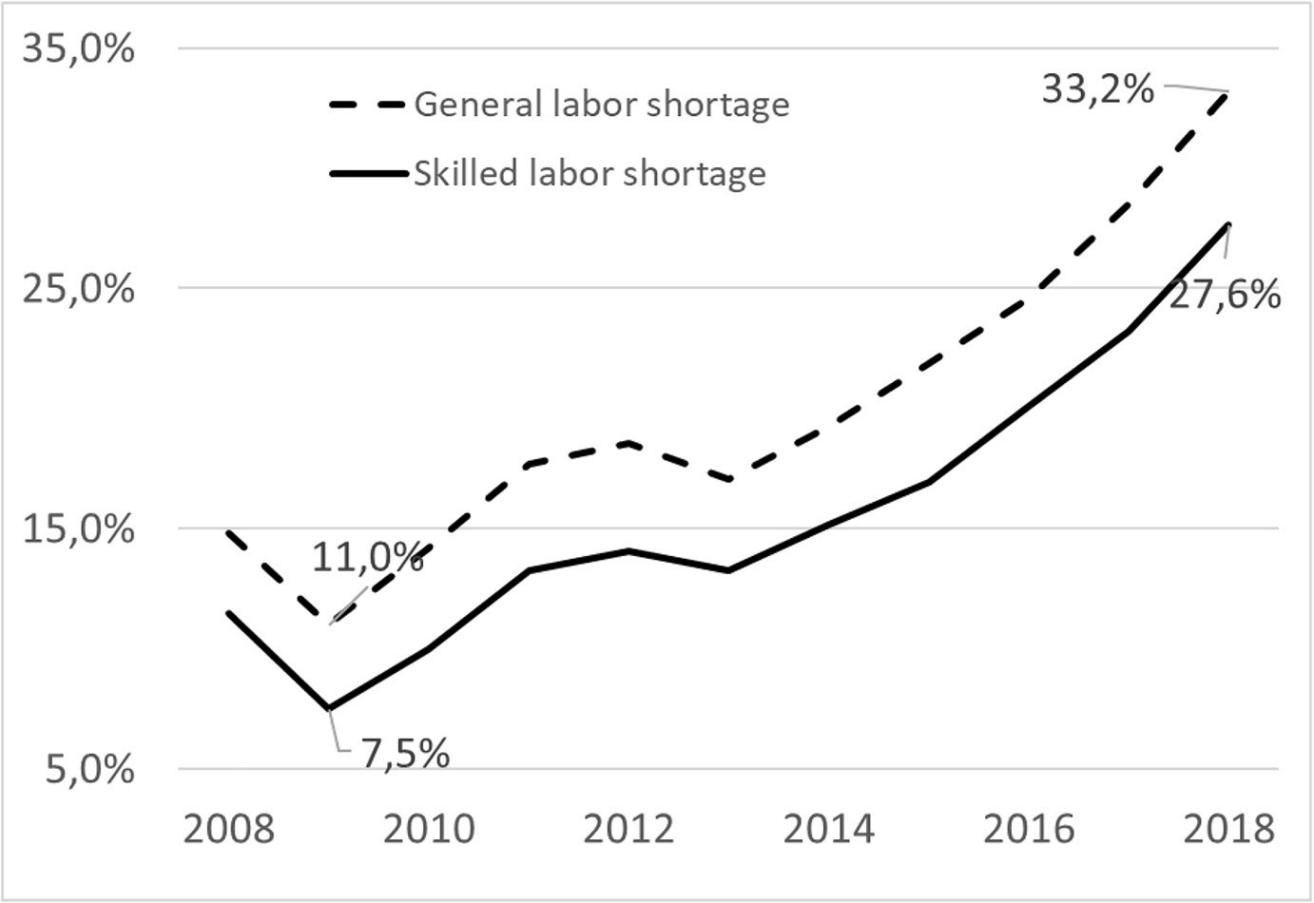
Share of firms reporting labor shortage. Source: IAB Establishment Panel 2008–2018

Figure 1 shows the problems with filling vacancies over the observed period from 2008 to 2018. After resolving these problems in the aftermath of the Great Recession in 2008/2009, the proportion of firms that could not fill all vacant positions until 2018 kept increasing. With the lowest value in 2009 at 11%, the share of firms with unfilled positions almost tripled over the period of observation. The corresponding figures for qualified employees were 7.5% and 27.6%, respectively. Thus, the numbers were lower than the 40% for 2018 cited in existing literature (Dettmann et al. 2019). The discrepancy may result from a different basis used to calculate the values in the latter study (“establishments with a need for skilled workers”) while all surveyed establishments are used in the present analysis. Furthermore, the shape of the two curves in Fig. 1 is almost similar. It can therefore be seen that the problem has increased significantly over time, affecting a considerable proportion of companies.

Moreover, I attempted to control for the influence of collective bargaining agreements. The data provides information on whether the firm is covered by an industrial-level or firm-level agreement or not bounded by a collective bargaining agreement. From this, the corresponding dummies can be derived. Additionally, the estimations used the interaction variables of both dummies indicating staffing problems and collective bargaining. Considering a flexible form of production function such as the translog, regressions should contain the corresponding variables for capital, labor, and output. As it was not possible to observe capital directly, the log of the running sum of expansion investments was used in its place. As a within transformation of the capital, the running sum of expansion investment gives the same result if the expansion investment is positive, the running sum of expansion investments should be a good instrument for capital in a panel regression. Also, the IAB Establishment Panel contains information on the revenue of enterprises in the year preceding the survey as a measure of output. Since the current study used this information, firms that did not report turnover, including banks, insurance companies and public administrations, were excluded from the database. However, turnover was not used directly here, instead, the value-added was used, thus eliminating intermediate materials from the turnover. Moreover, eight-firm size dummies were used to indicate the nonlinear influence of firm size on wages. Additional covariates from the IAB Establishment Panel were used to explain the wage level. These were the proportion of employees with a university degree; female employees; fixed-term employees; apprentices; employees subject to social insurance contributions; and dummies for West Germany; industry sectors; years; profitability; high level of competition; machinery condition; legal form; and whether the firm is a single establishment, managed by the owner, owned by foreigners or is exporting. Additionally, the Establishment History Panel contains information about the age and nationality of employees. The regressions, therefore, also included the proportion of employees who were younger than 25 and older than 50. The percentages of foreign workers from EU countries and outside the EU were also used here. The included Appendix presents the descriptive statistics for the principal variables (Appendix Table 6). As unobserved firm heterogeneities were considered, the model was estimated using a fixed-effects regression:

4 $$\ln \left({\mathrm{w}}_{\mathrm{i}\mathrm{t}}\right)=\kern0.5em {\upalpha \mathrm{c}}_{\mathrm{i}\mathrm{t}}+{\upgamma \mathrm{h}}_{\mathrm{i}\mathrm{t}}+{\upeta \mathrm{c}}_{\mathrm{i}\mathrm{t}}{\mathrm{h}}_{\mathrm{i}\mathrm{t}}+{\updelta}_{\mathrm{j}}{\mathrm{z}}_{\mathrm{j}\mathrm{it}}+{\mathrm{u}}_{\mathrm{i}}+{\mathrm{e}}_{\mathrm{i}\mathrm{t}},$$

with ln(w_it_) as the log of wages per capita of establishment i at time t. Variable c_it_ indicates coverage by a collective agreement, h_it_ are the dummies showing problems with filling positions, and c_it_h_it_ is the respective interaction variable. Variables z_jit_ represent additional covariates j. The α, γ, η, and δ_j_ are the corresponding parameters of the exogenous variables, u_i_ indicate unobserved firm heterogeneities, and e_it_ is the error term.

Another way to control for the unobserved fixed firm heterogeneities is to use first differences of the variables. Because the u_i_ are constant over time, Δu_i_ is zero and dropped from the estimation. The model then becomes:

5 $$\Delta \mathrm{ln}\left({\mathrm{w}}_{\mathrm{it}}\right)=\kern0.5em {\upalpha \Delta \mathrm{c}}_{\mathrm{it}}+{\upgamma \Delta \mathrm{h}}_{\mathrm{it}}+{\upeta \Delta \mathrm{c}}_{\mathrm{it}}{\Delta \mathrm{h}}_{\mathrm{it}}+{\updelta}_{\mathrm{j}}{\Delta \mathrm{z}}_{\mathrm{j}\mathrm{it}}+{\Delta \mathrm{e}}_{\mathrm{it}},$$

The simple use of Δc_it_ and Δh_it_ implies that the effects of changes in collective bargaining coverage and hiring problems on wages are symmetrical. If wages are downwardly rigid, then the introduction of collective bargaining agreements or the emergence of hiring problems should lead to different effects on wages than their abolition. Therefore, I used two dummies each in the model for the introduction or abolition of collective bargaining agreements and the occurrence or disappearance of restrictions on hiring. This also applies to the interaction variables. Moreover, dummies indicating continuous coverage of collective bargaining or maintaining the hiring problems are used to control for the differences compared with firms always without collective bargaining or hiring problems. The model then becomes:

6 $$\Delta \mathrm{ln}\left({\mathrm{w}}_{\mathrm{it}}\right)=\kern0.5em {\upalpha \Delta \mathrm{c}}_{\mathrm{ikt}}+{\upgamma \Delta \mathrm{h}}_{\mathrm{ikt}}+{\upeta \Delta \mathrm{c}}_{\mathrm{ikt}}{\Delta \mathrm{h}}_{\mathrm{ikt}}+{\updelta}_{\mathrm{j}}{\Delta \mathrm{z}}_{\mathrm{j}\mathrm{it}}+{\Delta \mathrm{e}}_{\mathrm{it}},$$

with k signaling the various change statuses in collective bargaining agreements and personnel problems.

As mentioned in the literature review, a low wage level induces a higher probability of observing hiring problems. Hence, it is possible that the observation of unfilled positions is endogenous to wages; therefore, the subsequent analysis applied a two-stage control function approach to account for this probable endogeneity (Wooldridge 2015). During the first stage, a regression of the potential endogenous variable was conducted using all other covariates of the structural model: This requires further use of additional variables as an instrument to explain why the hiring problems exist and fulfill the exclusion restriction. A strong instrument requires (partial) correlation with a potential endogenous variable. A correlation of the instrument and the endogenous variable of the estimation model at the second stage should go only via the channel of the instrumented variable. This first-stage regression is best described as a linear prediction or reduced form of estimation but not a structural model (Wooldridge 2010, 90). Possible instruments are the lagged indicator of hiring restrictions or the local unemployment rate. If hiring restrictions are due to a low wage level, then these should not occur before the decision to set wages. Hence, lagged staffing problems should not be endogenous to actual remunerations. Local unemployment rates indicate the tightness of labor markets which should influence the probability of observing hiring restrictions. Nevertheless, firms’ employment decisions are usually too small to influence local unemployment decisions on a NUTS III-level. The outcome for both instruments is similar at the first and the second stages of estimation and presented in the online supplement. Because lagged restrictions in hiring show a higher statistical validity than the local unemployment, the first is preferred to the latter. As information about staffing problems is a dummy variable, the first stage regression was conducted as a probit regression regarding the unobserved heterogeneity, according to the Mundlak/Chamberlain approach: This means that the regressions also contained the mean values of all time-variant exogenous variables multiplied by a dummy indicating the number of observations of each institution in the unbalanced panel (Wooldridge 2019). Here, the generalized residuals were calculated from the results of the probit regression and used as an additional covariate in the structural model of equation (1). If the parameter of this additional variable is significant, it is not possible to reject the influence of endogeneity.

## Regression results

Table 2 presents the results of the fixed-effects wage regressions for several specifications of labor shortage and collective bargaining agreements. A significant average wage premium of approximately 2.5% was found for workers in firms with a collective bargaining agreement. Similarly, employees in establishments with problems filling their vacancies received an average 1.4% higher wage than those in other establishments (column a). However, this only applies to establishments that have not concluded a collective bargaining agreement. Although the interaction variable between the two dummies showed a significant level of 10%, the size of the effect completely canceled out the wage effect that appeared when there were problems with filling the positions. Thus, in establishments covered by collective bargaining agreements, no further wage premium was observed when a shortage of labor occurred. When a dummy variable was used to represent shortages of skilled workers (column c), the results hardly changed. However, the interaction variable was now no longer statistically different from zero at a weak significance level. This may be due to downward rigidity in nominal wages, which is noticeable due to the short observation period. Subsequently, this was checked by the estimates in the first differences in Table 3. Overall, the estimates possibly confirm hypotheses i. to iii.

**Table 2:** Fixed effects wage regressions (Dependent variable: log. of wages per capita)

	(a) Restrictions in hiring workers	(b) Restrictions in hiring workers, endogeneity	(c) Restrictions in hiring skilled workers	(d) Restrictions in hiring workers, bargaining lev.
Generalized residual from first stage regression (control function)	–	0.005 (0.017)	–	
Collective bargaining agreement	0.026** (0.008)	0.031** (0.009)	0.025** (0.008)	
Industry-level collective bargaining agreement				0.028** (0.008)
Firm-level collective bargaining agreement.				0.018 (0.011)
Restrictions in hiring new workers	0.014** (0.005)	0.007 (0.029)	–	0.013* (0.005)
Restrictions in hiring new skilled workers	–	–	0.013** (0.005)	
Interaction variable between collective bargaining and hiring restrictions	−0.013^‡^ (0.008)	−0.009 (0.009)	−0.011 (0.008)	
Interaction variable between industry-level collective bargaining and hiring restrictions				−0.013^‡^ (0.008)
Interaction variable between firm-level collective bargaining and hiring restrictions				−0.012 (0.014)
Adj. R^2^	0.8669	0.8737	0.8669	0.8669
Observations (Establishments)	75,439 (22,087)	56,491 (15,993)	75,439 (22,087)	75,439 (22,087)

Source: IAB Establishment Panel 2008–2018.

Note: The model also includes the following dichotomous and auxiliary variables: Log. of running sum of expansion investment, log. of value added, shares of temporary employed, female workers, workers with a degree from university, workers subject to the German social security system, apprentices, foreign workers, workers younger than 25, workers older than 50, dummies for export, foreign ownership, individual ownership or partnership, single establishment, Western Germany and high competition, nine time dummies, 42 industry dummies, establishment size (six dummies), family management (two dummies), state of machinery (two dummies) and profitability (two dummies). Standard errors are adjusted for clustering on establishments. **; * and ‡ denote significance at the .01; .05 and .10 level, respectively

**Table 3:** First differences wage regressions (dependent variable: Δ of log. of establ. wages p. capita)

	(a) Restrictions in hiring workers	(b) Restrictions in hiring skilled workers
Collective bargaining agreement (ref.: no collective bargaining agreement)		
Introduction of a collective bargaining agreement	0.031* (0.013)	0.031* (0.013)
Abolition of a collective bargaining agreement	−0.011 (0.011)	−0.012 (0.011)
Maintaining of a collective bargaining agreement	-0.001 (0.003)	0.000 (0.003)
Restrictions in hiring new workers (ref.: no restrictions)		
Starting restrictions	0.019** (0.006)	0.018** (0.006)
Ending restrictions	−0.003 (0.007)	−0.001 (0.007)
Continuing restrictions	0.007 (0.005)	0.004 (0.005)
*Interaction variables*		
Introduction of a collective bargaining agreement•		
Starting restrictions	−0.017 (0.039)	−0.046 (0.042)
Ending restrictions	−0.028 (0.036)	−0.006 (0.033)
Continuing restrictions	−0.025 (0.030)	−0.020 (0.027)
Abolition of a collective bargaining agreement•		
Starting restrictions	−0.043 (0.031)	−0.036 (0.032)
Ending restrictions	0.038 (0.037)	0.037 (0.035)
Continuing restrictions	−0.018 (0.027)	−0.019 (0.029)
Maintaining of a collective bargaining agreement•		
Starting restrictions	−0.025** (0.009)	−0.028** (0.009)
Ending restrictions	0.005 (0.011)	−0.005 (0.011)
Continuing restrictions	−0.002 (0.007)	0.001 (0.008)
R^2^	0.0753	0.0752
F-Test (df1; df2)	27.08** (45; 13,262)	27.07** (45; 13,262)
Observations (Establishments)	46,242 (13,263)	46,242 (13,263)

Source: IAB Establishment Panel 2008–2018.

Note: The model also includes the following dichotomous and auxiliary variables: Log. of running sum of expansion investment, log. of value added, shares of temporary employed, female workers, workers with a degree from university, workers subject to the German social security system, apprentices, foreign workers, workers younger than 25, workers older than 50, dummies for export, foreign ownership, individual ownership or partnership, single establishment and high competition, establishment size (six dummies), family management (two dummies), state of machinery (two dummies) and profitability (two dummies). Standard errors are adjusted for clustering on establishments. **; * and ‡ denote significance at the .01; .05 and .10 level, respectively

Column (b) contains the estimates that account for the possible endogeneity of job-filling problems. Appendix Table 5 in the appendix presents the average partial effects of the first stage estimates. The additional instruments described above are expected to strongly correlate with the job-filling problems. The significant negative parameter estimates indicate a decreasing probability for observing hiring restrictions if these problems were present in the year before. A high correlation is sufficient, and the outcome of an F-test (F(2, 401) = 146.17**) confirmed a relevant significance of the dummy. In addition to these variables, a high share of trainees, employees subject to social security contributions, a high level of competition, and a location in western Germany, reduce the probability of observing problems with staffing. By contrast, the Log. of running sum of investments, the Log. of values added, the share of temporary employees, and higher profitability increased the likelihood of such an observation.

The generalized residual was then calculated from the estimates and used as an additional covariate in the wage estimates. However, since the estimated values were insignificant, the assumption can be rejected. Although the parameter for staffing problems was also insignificant in each case, t-tests showed no systematic differences from the estimated values in column (a). Even in cases where not all positions for qualified employees could be filled, it was still not possible to detect the endogeneity of the staffing problems. Therefore, the results are not presented here.

Column (d) contains the results involving the distinction between firm-level or industry-level collective bargaining agreements. Here, no structural differences emerged between the two types of collective bargaining agreements. The estimated values were similar to those from the previous regressions. Although the parameters for the influence of firm-level collective agreements became insignificant, the values were similar to those in firms with agreements on the industry level. This could be due to the small number of cases that enter this type of collective agreement. Only 5% of the observations had a firm-level collective agreement. Since there were only slight differences in further regressions, additional estimates for the types of collective agreements are not presented here. However, the results are available on request from the author.

As mentioned above, the (almost) insignificant values of the interaction variable between the collective bargaining coverage and staffing problems may indicate downward rigidities in nominal wages: the occurrence of staffing problems or a newly concluded collective bargaining agreement led to wage increases while resolving the staffing problems or leaving a collective bargaining coverage did not lead to wage decreases. Therefore, Table 3 presents first-difference estimates, including the firm-specific fixed effects, and represents the different states of the collective bargaining coverage and staffing problems. Since no endogeneity was detected from the results in Table 2, only estimates without the generalized residual are presented. As usual, estimations in the first differences reduced the number of observations. Although the efficiency also decreased, the outcomes expected should be consistent. It can be seen that the conclusion of a collective bargaining agreement led to an average salary increase of 3.1% for employees. By contrast, the abolition of collective bargaining led to no significant change in pay. The insignificant estimation result for continuing the collective bargaining coverage indicates there were no continuously strong wage increases as a result of the collective bargaining. The situation is similar for the problems of filling all vacancies. Here, wages rose by just under 2%, if not enough workers can be recruited. Otherwise, the effects were insignificant here as well. The same was true for most of the interaction variables. Only the effects for newly observed problems of filling positions in establishments with collective bargaining agreements were highly significant and canceled out the positive effect on compensation. Table 4 contains the results for a test of joint significance of both variables, indicating that the added effect is not statistically different from zero.

**Table 4:** T-test on the effect of starting hiring restrictions in establishments with a collective bargaining agreement according to the estimates in Table 3

	(a) Restrictions in hiring workers	(b) Restrictions in hiring skilled workers
Started hiring restrictions in establishments in collective bargaining agreement	−0.007 (0.007)	−0.009 (0.007)

Source: IAB Establishment Panel 2008–2018.

Note: Test of joint significance of estimates of the variables “starting restrictions” and the interaction variable “maintaining of a collective bargaining agreement”• “starting restrictions”

Again, this confirms the hypothesis that a wage premium because of staffing problems is paid only when there is no collective bargaining coverage. If labor shortage is related to the business cycle, the outcomes confirm the previous observation of a countercyclical development of the wage premium in firms with a collective bargaining agreement (Bratsberg and Ragan Jr. 2002; Blanchflower and Bryson 2004). In contrast to the previous explanations, it is not the behavior of the unions that is responsible for this, but of those companies not bound by collective agreements, which make higher wage offers. In addition to the first three hypotheses, the first difference estimates also failed to reject hypothesis iv. Moreover, unions cannot create further wage increases if a collective bargaining agreement is introduced in firms with labor shortages.

## Summary

This study examines the relationship between wage levels and problems with filling vacancies. Existing literature discusses various ways of dealing with such a situation. In addition to more flexible work processes, a substitution of labor, or the employment of workers who do not (yet) have the necessary qualifications, companies could also enter into wage competition with other firms to attract suitable workers. In such a case, a shortage of skilled labor would lead to higher average wages for employees. This corresponds with results from representative firm surveys where firms stated that applicants demanded too high a wage; thus, explains why the occupations affected are not primarily highly skilled, such as nurses. However, such models of wage competition assume – just as the monopsony model does – that firms want to pay a wage that equals the reservation wage and, at most, wages that equals the marginal revenue of labor. By contrast, models of collective bargaining assume that the wage level is pushed to the marginal revenue of labor and may even exceed it in the case of rent sharing. If establishments compete over wage levels when filling jobs, then establishments bound by collective bargaining agreements should have an advantage because they pay a higher wage than establishments not bound by collective agreements.

Based on the review of the existing literature and theoretical considerations, four hypotheses were derived, all empirically tested using a representative panel data set of German establishments from 2008 to 2018. The multivariate wage regressions used the log of firm-level average wages, accounting for firm-specific heterogeneities and controlling for the influence of labor shortages and coverage by collective bargaining agreements through appropriate covariates. It was not possible to confirm the endogeneity of labor shortages. The results largely supported the established hypotheses. In summary, the collective bargaining agreement and existing staffing problems led to a wage premium, although the latter is only paid in firms not bound by a collective bargaining agreement. Thus, there is no additional wage premium for firms that participate in collective agreements when skill shortages occur, and then, the difference in pay between the two types of firms becomes smaller. This confirms the countercyclical movements of the wage premium from collective agreements, but not due to the strength of union bargaining power. Due to downward rigidities in nominal wages, this effect can be observed, especially when staffing problems occur for the first time.

One caveat to the analysis arises from using establishment data and average wages. The human capital theory defines an employee’s pay in terms of individual factors, such as education and work experience. These factors may only reflect a limited extent of the company data on the skill structure. Therefore, this could also be the starting point for further studies to confirm the present results with panel data for individual employees.

### Supplementary Information

ESM 1 (DOCX 27 kb)

## Appendix

**Table 5:** Average partial effects of Mundlak/Chamberlain panel probit regressions about restrictions in hiring new workers (dependent variable = 1 if firm reports restrictions)

	(a) Firm reports restrictions in hiring new workers	(b) firm reports restrictions in hiring new skilled workers
Lagged hiring restrictions	−0.043** (0.004)	−0.046** (0.003)
Log. of running sum of expansion investments	0.004** (0.001)	0.003** (0.001)
Collective bargaining agreement	−0.002 (0.007)	−0.004 (0.006)
Log. of valued added	0.007* (0.004)	0.006‡ (0.003)
Share of workers with a degree from university	0.021 (0.018)	0.007 (0.017)
Share of female workers	0.023 (0.017)	0.035* (0.015)
Share of temporary employed	0.052** (0.019)	0.058** (0.018)
Share of workers subject to the German social security system	−0.086** (0.016)	−0.081** (0.015)
Share of apprentices	−0.055‡ (0.032)	−0.024 (0.030)
Share of EU foreign workers	−0.043 (0.028)	−0.016 (0.025)
Share of non-EU foreign workers	0.000 (0.029)	−0.019 (0.026)
Share of workers older than 50	0.053** (0.013)	0.044** (0.013)
Share of workers younger than 25	0.010 (0.019)	0.013 (0.017)
Exporting firm	0.001 (0.008)	0.001 (0.007)
Foreign ownership	0.005 (0.014)	−0.005 (0.014)
Individually owned or partnership	0.004 (0.016)	0.007 (0.015)
Single establishment	−0.002 (0.009)	−0.002 (0.008)
High competition	−0.010** (0.004)	−0.010** (0.004)
Western Germany	−0.005* (0.002)	−0.003 (0.002)
Profitability (ref.: low profitability)		
high	0.020** (0.005)	0.026** (0.005)
average	0.007 (0.004)	0.012** (0.004)
Log. pseudolikelihood	−18,478.229	−16,202.855
Pseudo-R^2^	0.4099	0.4228
Wald-Test χ^2^(df.)	23,426.93** (403)	20,504.18** (403)
Observations (Establishments)	62,879 (17,297)	62,879 (17,297)

Source: IAB Establishment Panel 2008–2018.

Note: The model also includes the following dichotomous and auxiliary variables: family management (two dummies) and state of machinery (two dummies), nine time dummies, establishment size (six dummies), 42 industry dummies. The Chamberlain/Mundlak approach for unbalanced panels requires including the means of the time-varying covariates and an indicator that identifies the number of observations of each unit respectively the interactions of both in the regression (Wooldridge 2019). Standard errors are adjusted for clustering on establishments. **; * and ‡ denote significance at the .01; .05 and .10 level, respectively

**Table 6:** Descriptive statistics of variables in sample

Variable	Observations	Mean	Std. dev.	Minimum	Maximum
Existing working time accounts	106,222	0.461	0.498	0	1
Planned working time accounts	106,222	0.018	0.132	0	1
Log. of running sum of expansion investments	106,737	5.776	5.999	0	20.690
Coverage by a collective bargaining agreement (industry level)	106,405	0.279	0.449	0	1
Coverage by a collective bargaining agreement (firm level)	106,405	0.050	0.217	0	1
Log. of value added	93,327	13.101	2.137	3.792	23.044
Share of workers with university degree	105,415	0.079	0.174	0	2
Share of female workers	106,707	0.391	0.308	0	1
Share of fixed-term workers	106,117	0.044	0.118	0	1
Share of workers subject to social insurance contributions	106,737	0.709	0.297	0	1
Share of apprentices	105,420	0.041	0.084	0	0.795
Share of workers from EU countries	106,737	0.146	0.329	0	1
Share of workers from non-EU countries	106,737	0.148	0.330	0	1
Share of workers older than 50	106,737	0.426	0.319	0	1
Share of workers younger than 25	106,737	0.212	0.326	0	1
Share of exporting firms	105,987	0.239	0.426	0	1
Share of foreign owned firms	103,567	0.053	0.224	0	1
Share of individually owned firms or partnerships	106,376	0.431	0.495	0	1
Share of single establishment firms	105,663	0.829	0.376	0	1
High level of competition dummy	106,589	0.385	0.486	0	1
Western Germany (dummy)	106,737	0.570	0.495	0	1
Exclusively managed by owners	102,158	0.728	0.445	0	1
Partly managed by owners	102,158	0.069	0.254	0	1
Very good / good condition of machinery	106,502	0.156	0.363	0	1
Average condition of machinery	106,502	0.478	0.500	0	1
High profitability	106,373	0.477	0.499	0	1
Average profitability	106,373	0.308	0.461	0	1

Source: IAB Establishment Panel 2008–2018

## References

[CR1] Addison JT, Bryson A, Teixeira P, Pahnke A, Bellmann L (2013). The Extent of Collective Bargaining and Workplace Representation: Transitions between States and their Determinants. A Comparative Analysis of Germany and Great Britain. Scottish Journal of Political Economy.

[CR2] Addison J, Teixeira P, Evers K, Bellmann L (2014). Indicative and updated estimates of the collective bargaining premium in Germany. Industrial Relations: A Journal of Economy and Society.

[CR3] Ainslie G, Haslam N, Loewenstein G, Elster J (1992). Hyperbolic discounting. *Choice over time*.

[CR4] Amlinger M (2014) Lohnhöhe und Tarifbindung: Bestimmungsfaktoren der individuellen Verdiensthöhe. WSI Report No. 20

[CR5] Andrews MJ, Bradley S, Stott D, Upward R (2008). Successful employer search? An empirical analysis of vacancy duration using micro data. Economica.

[CR6] Banfi S, Villena-Roldan B (2019). Do high-wage jobs attract more applicants? Directed search evidence from the online labor market. Journal of Labor Economics.

[CR7] Barron JM, Berger MC, Black DA (1997). Employer search, training, and vacancy duration. Economic Inquiry.

[CR8] Blanchflower DG, Bryson A (2004). What effect do unions have on wages now and would Freeman and Medoff be surprised?. Journal of Labor Research.

[CR9] Bossler M, Kubis A, Küfner B, Popp M (2021) The IAB Job Vacancy Survey: Establishment survey on labour demand and recruitment processes, waves 2000 to 2018 and subsequent quarters 2006 to 2019. FDZ-Datenreport 09/2021, Nuremberg

[CR10] Braakmann N and Brandl B (2020) The Performance of Collective and Individual Bargaining: A Comprehensive and Granular Analysis of Different Bargaining Systems on Company Productivity. Int Labour Rev 160(1), 43–64

[CR11] Brändle T, Grunau P, Haylock M, and Kampkötter P (2020) Recruitment strategies and match quality-new evidence from representative linked employer-employee data. University of Tübingen Working Papers in Business and Economics No. 134

[CR12] Branten E, Lamo A, Room T (2018) Nominal wage rigidity in the EU countries before and after the Great Recession: evidence from the WDN surveys. ECB Working Paper No. 2159

[CR13] Bratsberg B, Ragan JF (2002). Changes in the union wage premium by industry. ILR Review.

[CR14] Brenčič V (2010). Do employers respond to the costs of continued search?. Oxford Bulletin of Economics and Statistics.

[CR15] Brenzel H, Gartner H, C., and Schnabel. (2014). Wage bargaining or wage posting? Evidence from the employers' side. Labour Economics.

[CR16] Burdett K, Mortensen DT (1998). Wage differentials, employer size, and unemployment. International Economic Review.

[CR17] Cahuc P, Carcillo S, and Zylberberg A (2014) Labor economics (2^nd^ ed.). MIT Press

[CR18] Cahuc P, Postel-Vinay F, Robin JM (2006). Wage bargaining with on-the-job search: Theory and evidence. Econometrica.

[CR19] Carrillo-Tudela C, Gartner H, and Kaas L (2020) Recruitment Policies, Job-Filling Rates and Matching Efficiency. IZA Discussion Paper No. 13240

[CR20] Dettmann E, Fackler D, Mueller S, Neuschäffer G, Slavtchev V, Leber U, and Schwengler B (2019) Fehlende Fachkräfte in Deutschland-Unterschiede in den Betrieben und mögliche Erklärungsfaktoren: Ergebnisse aus dem IAB-Betriebspanel 2018. *IAB-Forschungsbericht* No. 10/2019

[CR21] Eberle J and Schmucker A (2017) The Establishment History Panel–Redesign and Update 2016. J Econ Stat (Jahrbücher für Nationalökonomie und Statistik) 237(6), 535–547

[CR22] Ellguth P, Kohaut S (2019). Tarifbindung und betriebliche Interessenvertretung: Ergebnisse aus dem IAB-Betriebspanel 2018. WSI-Mitteilungen.

[CR23] Ehrenfried F, Holzner C (2019). Dynamics and endogeneity of firms’ recruitment behaviour. Labour Economics.

[CR24] Federal Employment Agency (2020) *Fachkräfteengpassanalyse 2019*. Berichte: Blickpunkt Arbeitsmarkt. October 2020, Nuremberg

[CR25] Fischer G, Janik F, Müller D, Schmucker A (2009). The IAB Establishment Panel – Things Users Should Know. Journal of Applied Social Science Studies.

[CR26] Freeman RB, Medoff JL (2018). *What Do Unions Do?*.

[CR27] Gavazza A, Mongey S, Violante GL (2018). Aggregate recruiting intensity. American Economic Review.

[CR28] Gürtzgen N (2016). Estimating the wage premium of collective wage contracts: Evidence from longitudinal linked employer–employee data. Industrial Relations: A Journal of Economy and Society.

[CR29] Hirsch B, Mueller S (2020). Firm wage premia, industrial relations, and rent sharing in Germany. ILR Review.

[CR30] Jung S, Schnabel C (2011). Paying more than necessary?. The wage cushion in Germany. Labour.

[CR31] Kaas L, Kircher P (2015). Efficient firm dynamics in a frictional labor market. American Economic Review.

[CR32] Leduc S, Liu Z (2020). The weak job recovery in a macro model of search and recruiting intensity. American Economic Journal: Macroeconomics.

[CR33] Leifheit A (2018) Many job openings, few job seekers: SMEs expect a shortage of skilled workers. KfW Research Focus on Economics No. 232

[CR34] Lochner B, Merkl C, Stüber H, Gürtzgen N (2021). Recruiting intensity and hiring practices: Cross-sectional and time-series evidence. Labour Economics.

[CR35] Manning A, Ashenfelter O, Card D (2011). Imperfect competition in the labor market. *Handbook of Labor Economics*.

[CR36] Marinescu I, Wolthoff R (2020). Opening the black box of the matching function: The power of words. Journal of Labor Economics.

[CR37] Morin A (2017). Cyclicality of wages and union power. Labour Economics.

[CR38] Peng F, Anwar S, Kang L (2020). Job Movement and Real Wage Flexibility in Eastern and Western Parts of Germany. Journal of Economics and Finance.

[CR39] Schaal E (2017). Uncertainty and unemployment. Econometrica.

[CR40] Sedláček P (2014). Match efficiency and firms' hiring standards. Journal of Monetary Economics.

[CR41] Wooldridge JM (2010). *Econometric Analysis of Cross Section and Panel Data*.

[CR42] Wooldridge JM (2015). Control function methods in applied econometrics. Journal of Human Resources.

[CR43] Wooldridge JM (2019). Correlated Random Effects Models with Unbalanced Panels. Journal of Econometrics.

