# Supplementary material for: Shortage of Skilled Labor, Unions and the Wage Premium: A Regression Analysis with Establishment Panel Data for Germany
Source: J Labor Res. 2022 Jun 22;43(2):239–59. doi: 10.1007/s12122-022-09334-1 (PMC9214463; doi:10.1007/s12122-022-09334-1)
Supplement: Supplementary file 1 — (DOCX 27 kb) [file 12122_2022_9334_MOESM1_ESM.docx]

## **Supplement**

## *Table S.1: Average partial effects of Mundlak/Chamberlain panel probit regressions about restrictions in hiring new workers (dependent variable = 1 if firm reports restrictions)*

|  | Firm reports restrictions in hiring new workers | | firm reports restrictions in hiring new skilled workers | |
| --- | --- | --- | --- | --- |
|  | (a) | (b) | (c) | (d) |
| Lagged restrictions in hiring new workers |  | -0.043** (0.004) |  | -0.046** (0.003) |
| Log. of local unemployment rate (NUTS III) | -0.038* (0.017) |  | -0.039* (0.015) |  |
| Log. of running sum of expansion investments | 0.001* (0.001) | 0.004** (0.001) | 0.001* (0.001) | 0.003** (0.001) |
| Dummy if firms participate in a collective bargaining agreement | 0.005 (0.007) | -0.002 (0.007) | 0.002 (0.007) | -0.004 (0.006) |
| Log. of valued added | 0.003 (0.004) | 0.007* (0.004) | 0.004 (0.004) | 0.006‡ (0.003) |
| Share of workers with a degree from university | 0.041* (0.019) | 0.021 (0.018) | 0.026 (0.019) | 0.007 (0.017) |
| Share of female workers | 0.006 (0.021) | 0.023 (0.017) | 0.025 (0.020) | 0.035* (0.015) |
| Share of temporary employed | 0.065** (0.021) | 0.052** (0.019) | 0.064** (0.020) | 0.058** (0.018) |
| Share of workers subject to the German social security system | -0.079** (0.021) | -0.086** (0.016) | -0.083** (0.020) | -0.081** (0.015) |
| Share of apprentices | -0.093* (0.039) | -0.055‡ (0.032) | -0.055 (0.036) | -0.024 (0.030) |
| Share of EU foreign workers | -0.050 (0.035) | -0.043 (0.028) | -0.047 (0.033) | -0.016 (0.025) |
| Share of non-EU foreign workers | -0.023 (0.038) | 0.000 (0.029) | -0.047 (0.039) | -0.019 (0.026) |
| Share of workers older than 50 | -0.001 (0.016) | 0.053** (0.013) | 0.000 (0.016) | 0.044** (0.013) |
| Share of workers younger than 25 | 0.046* (0.021) | 0.010 (0.019) | 0.054** (0.019) | 0.013 (0.017) |
| Dummy if firm is exporting | 0.009 (0.008) | 0.001 (0.008) | 0.004 (0.008) | 0.001 (0.007) |
| Dummy if foreign ownership | 0.002 (0.016) | 0.005 (0.014) | -0.003 (0.015) | -0.005 (0.014) |
| Dummy if individually owned or partnership | -0.004 (0.019) | 0.004 (0.016) | -0.005 (0.018) | 0.007 (0.015) |
| Dummy if single establishment | -0.007 (0.009) | -0.002 (0.009) | -0.009 (0.008) | -0.002 (0.008) |
| Dummy if high competition | -0.016** (0.004) | -0.010** (0.004) | -0.013** (0.004) | -0.010** (0.004) |
| Dummy if Western Germany | -0.041** (0.006) | -0.005* (0.002) | -0.043** (0.005) | -0.003 (0.002) |
| Profitability (ref.: low profitability) |  |  |  |  |
| high | 0.022** (0.005) | 0.020** (0.005) | 0.028** (0.005) | 0.026** (0.005) |
| average | 0.007 (0.005) | 0.007 (0.004) | 0.013** (0.005) | 0.012** (0.004) |
| Log. pseudolikelihood | -31,942.293 | -18,478.229 | -27,913.946 | -16,202.855 |
| Pseudo-R² | 0.1374 | 0.4099 | 0.1551 | 0.4228 |
| Wald-Test χ²(df.) | 5,411.96** (437) | 23,426.93** (403) | 5441.44** (437) | 20,504.18** (403) |
| Observations (Establishments) | 71,439 (21,629) | 62,879 (17,297) | 71,439 (21,629) | 62,879 (17,297) |

Source: IAB Establishment Panel 2008–2018.

Note: The model also includes the following dichotomous and auxiliary variables: family management (two dummies) and state of machinery (two dummies), nine time dummies, establishment size (six dummies), 42 industry dummies. The Chamberlain/Mundlak approach for unbalanced panels requires including the means of the time-varying covariates and an indicator that identifies the number of observations of each unit respectively the interactions of both in the regression (Wooldridge 2019). Standard errors are adjusted for clustering on establishments. **; * and ‡ denote significance at the .01; .05 and .10 level, respectively.

## *Table S.2: Fixed effects wage regressions (Dependent variable: log. of wages per capita)*

|  | (a) Restrictions in hiring workers | (b) Restrictions in hiring skilled workers | (c)  Restrictions in hiring workers | (d)  Restrictions in hiring skilled workers |
| --- | --- | --- | --- | --- |
|  | (Instrument: Log of local unemployment rate, NUTS III) | | (Instrument: Lagged restrictions in hiring workers) | |
| Generalized residual from first stage regression (control function) | -0.019 (0.028) | -0.026 (0.025) | 0.005 (0.017) | 0.000 (0.016) |
| Dummy if firms participate in a collective bargaining agreement | 0.020* (0.008) | 0.020* (0.008) | 0.031** (0.009) | 0.031** (0.009) |
| Dummy if firm reports restrictions in hiring new workers | 0.045 (0.048) |  | 0.007 (0.029) |  |
| Dummy if firm reports restrictions in hiring new skilled workers |  | 0.057 (0.043) |  | 0.015 (0.028) |
| Interaction variable between collecting bargaining and restrictions of hiring | -0.015 (0.008) | -0.015 (0.008) | -0.009 (0.009) | -0.010 (0.009) |
| Adj. R² | 0.8701 | 0.8701 | 0.8737 | 0.8737 |
| Observations (Establishments) | 66,562 (20,333) | 66,562 (20,333) | 56,491 (15,993) | 56,491 (15,993) |

Source: IAB Establishment Panel 2008–2018.

Note: The model also includes the following dichotomous and auxiliary variables: Log. of running sum of expansion investment, log. of value added, shares of temporary employed, female workers, workers with a degree from university, workers subject to the German social security system, apprentices, foreign workers, workers younger than 25, workers older than 50, dummies for export, foreign ownership, individual ownership or partnership, single establishment, Western Germany and high competition, nine time dummies, 42 industry dummies, establishment size (six dummies), family management (two dummies), state of machinery (two dummies) and profitability (two dummies). Standard errors are adjusted for clustering on establishments. **; * and ‡ denote significance at the .01; .05 and .10 level, respectively.

## *Table S.3: First differences wage regressions (dependent variable: Δ of log. of establ. wages p. capita)*

|  | (a) Restrictions in hiring workers | (b)  Restrictions in hiring skilled workers | (a) Restrictions in hiring workers | (b)  Restrictions in hiring skilled workers |
| --- | --- | --- | --- | --- |
|  | (Instrument: Log of local unemployment rate, NUTS III) | | (Instrument: Lagged restrictions in hiring workers) | |
| Δ Generalized residual from first stage regression (control function) | 0.027 (0.020) | 0.024 (0.018) | 0.025 (0.015) | 0.026 (0.014) |
| Collective bargaining agreement (ref.: no collective bargaining agreement) |  |  |  |  |
| Introduction of a collective bargaining agreement | 0.035* (0.014) | 0.033* (0.014) | 0.036* (0.017) | 0.034* (0.016) |
| Abolition of a collective bargaining agreement | -0.011 (0.012) | -0.011 (0.011) | -0.009 (0.013) | -0.012 (0.013) |
| Maintaining of a collective bargaining agreement | -0.002 (0.003) | -0.001 (0.003) | -0.002 (0.003) | -0.002 (0.003) |
| Restrictions in hiring new workers (ref.: no restrictions) |  |  |  |  |
| Starting restrictions | -0.032 (0.034) | -0.027 (0.031) | -0.023 (0.026) | -0.027 (0.024) |
| Ending restrictions | 0.045 (0.035) | 0.040 (0.032) | 0.036 (0.028) | 0.038 (0.024) |
| Continuing restrictions | 0.005 (0.005) | 0.003 (0.006) | 0.002 (0.005) | 0.003 (0.006) |
| *Interaction variables* |  |  |  |  |
| Introduction of a collective bargaining agreement• |  |  |  |  |
| Starting restrictions | -0.032 (0.044) | -0.066 (0.046) | -0.003 (0.036) | -0.013 (0.037) |
| Ending restrictions | -0.038 (0.041) | -0.009 (0.036) | -0.063 (0.047) | -0.013 (0.038) |
| Continuing restrictions | -0.034 (0.030) | -0.015 (0.024) | 0.011 (0.030) | -0.006 (0.025) |
| Abolition of a collective bargaining agreement• |  |  |  |  |
| Starting restrictions | -0.029 (0.033) | -0.037 (0.035) | -0.048 (0.037) | -0.027 (0.037) |
| Ending restrictions | 0.055 (0.043) | 0.056 (0.040) | 0.013 (0.038) | 0.011 (0.032) |
| Continuing restrictions | -0.013 (0.028) | -0.009 (0.031) | -0.013 (0.032) | -0.014 (0.036) |
| Maintaining of a collective bargaining agreement• |  |  |  |  |
| Starting restrictions | -0.026** (0.010) | -0.028** (0.010) | -0.021* (0.010) | -0.022* (0.010) |
| Ending restrictions | 0.005 (0.011) | -0.004 (0.011) | 0.005 (0.012) | 0.001 (0.012) |
| Continuing restrictions | 0.000 (0.008) | 0.001 (0.008) | 0.005 (0.008) | 0.004 (0.009) |
| R² | 0.0705 | 0.0705 | 0.0673 | 0.0672 |
| Observations (Establishments) | 40,103 (13,263) | 40,103 (13,263) | 35,761 (10,330) | 35,761 (10,330) |

Source: IAB Establishment Panel 2008–2018.

Note: The model also includes the following dichotomous and auxiliary variables: Log. of running sum of expansion investment, log. of value added, shares of temporary employed, female workers, workers with a degree from university, workers subject to the German social security system, apprentices, foreign workers, workers younger than 25, workers older than 50, dummies for export, foreign ownership, individual ownership or partnership, single establishment and high competition, establishment size (six dummies), family management (two dummies), state of machinery (two dummies) and profitability (two dummies). Standard errors are adjusted for clustering on establishments. **; * and ‡ denote significance at the .01; .05 and .10 level, respectively.
